# Symptoms of anxiety and depression in lesbian couples treated with donated sperm: a descriptive study

**DOI:** 10.1111/1471-0528.12214

**Published:** 2013-03-14

**Authors:** C Borneskog, G Sydsjö, C Lampic, M Bladh, AS Svanberg

**Affiliations:** a Department of Women's and Children's Health, Uppsala UniversityUppsala, Sweden; b Obstetrics and Gynaecology, Department of Clinical and Experimental Medicine, Faculty of Health Sciences, Linköping UniversityLinköping, Sweden; c Department of Gynaecology and Obstetrics in Linköping, County Council of ÖstergötlandLinköping, Sweden; d Department of Neurobiology, Care Sciences and Society, Karolinska InstitutetHuddinge, Sweden

**Keywords:** Anxiety and depression, assisted reproduction, lesbian

## Abstract

**Objective:**

To investigate symptoms of anxiety and depression in lesbian couples undergoing assisted reproductive treatment (ART), and to study the relationship of demographic data, pregnancy outcome and future reproductive plans with symptoms of anxiety and depression.

**Design:**

Descriptive, a part of the prospective longitudinal ‘Swedish study on gamete donation’.

**Setting:**

All university clinics in Sweden performing gamete donation.

**Population:**

A consecutive sample of 214 lesbian couples requesting assisted reproduction, 165 of whom participated.

**Methods:**

Participants individually completed three study-specific questionnaires and the Hospital Anxiety and Depression Scale (HADS): time point 1 (T1), at commencement of ART; time point 2 (T2), approximately 2 months after treatment; and time point 3 (T3), 2–5 years after first treatment.

**Main outcome measures:**

Anxiety and depression (HADS), pregnancy outcome and future reproductive plans.

**Results:**

The vast majority of lesbian women undergoing assisted reproduction reported no symptoms of anxiety and depression at the three assessment points. A higher percentage of the treated women, compared with the partners, reported symptoms of anxiety at T2 (14% versus 5%, *P* = 0.011) and T3 (10% versus 4%, *P* = 0.018), as well as symptoms of depression at T2 (4% versus 0%, *P* = 0.03) and T3 (3% versus 0%, *P* = 0.035). The overall pregnancy outcome was high; almost three-quarters of lesbian couples gave birth 2–5 years after sperm donation treatments. Open-ended comments illustrated joy and satisfaction about family building.

**Conclusion:**

Lesbian women in Sweden reported good psychological health before and after treatment with donated sperm.

## Introduction

Lesbian women conceiving through donor insemination are of particular interest as lesbian couples represent a growing group of patients in obstetric and maternity health care.^1^

Anxiety and depressive disorders are common in fertile women^2^ and in the general population, and are two to three times as common in women than in men.^2^ In a Scandinavian population, the 12-month prevalence of major depression varies between 4.5 and 9.7% in women and 3 and 4.1% in men.^3^,^4^ Previous research has described greater psychological morbidity in lesbian women compared with heterosexual women,^5^–^7^ mainly as a consequence of minority stress.^8^–^12^ Perceived social support,^13^ relationship satisfaction,^1^,^14^ disclosure of sexual orientation^15^,^16^ and the unique role of the co-mother^17^,^18^ are other factors that have been reported to impact on anxiety and depressive disorders in lesbian women. Although available research is limited,^19^ the perinatal period has been identified as a time of increased risk of psychiatric illness in women,^19^–^21^ and women with previous mental health problems have been found to be more vulnerable to maternal distress^19^,^21^,^22^ and postpartum depression.^14^,^19^,^21^

Symptoms of anxiety and depression in heterosexual women undergoing *in vitro* fertilisation (IVF) treatment have frequently been reported^23^,^24^ and, although many of the aspects of conceiving and parenthood are shared between lesbian and heterosexual women, lesbian women may differ from heterosexual women with regard to a number of variables that have been associated with perinatal mental health.^9^,^25^ To our knowledge, long-term follow-up of anxiety and depressive symptoms in lesbian couples participating in assisted reproduction through donor sperm insemination, resulting in pregnancy and childbirth, has not been studied. The aim of this study was to investigate symptoms of anxiety and depression in lesbian couples during a 2-year period after sperm donation treatment, and to study the relationship of demographic background data (educational level and previous children), pregnancy outcome after sperm donation treatment and future reproductive plans with symptoms of anxiety and depression.

## Materials and methods

### Sample and procedure

The Swedish study on gamete donation is a prospective longitudinal study of donors and recipients of donated gametes. The multicentre study includes all fertility clinics performing gamete donation in Sweden, at the university hospitals in Stockholm, Gothenburg, Uppsala, Umeå, Linköping, Örebro and Malmö. This study presents data from lesbian couples using donor sperm to conceive. During 2005–2008, a consecutive cohort of lesbian couples at the commencement of assisted reproductive treatment (ART) were approached for participation, and data were collected consecutively during 2005–2011. The first questionnaires were handed out to the couples by staff at the fertility clinic. The second and third questionnaires were distributed by mail, together with a prepaid return envelope and a covering letter stating the purpose of the study and guaranteeing confidentiality. Nonresponders were sent two reminders.

Participants individually completed questionnaires at three time points: at the commencement of treatment (T1); approximately 2 months after the first treatment (T2); and 2–5 years after the first treatment (T3). As the third questionnaire aimed to investigate psychosocial aspects in the family when the donor offspring were around 12 months of age, the third questionnaire was sent out when the child was between 12 and 18 months of age. Because of this, T3 varies within the couples and the responses from T3 were collected 2–5 years after the first treatment (T1). Couples that did not complete at least one round of treatment (which included one sperm insemination treatment or one cycle of regular IVF) were excluded from the study. Couples who did not speak or read Swedish were also excluded.

#### Lesbian couples treated with donor sperm insemination and/or IVF with donated sperm

A total of 214 lesbian couples (428 individuals) who started treatment with sperm donation were approached to participate in the study; of these, 165 couples (330 individuals) agreed to participate (77% response rate). Reasons for nonparticipation were as follows: did not want to participate (*n* = 54), treatment discontinuation (*n* = 34) or not stated (*n* = 10).

Medical data were collected from 160 of the treated lesbian women (five missing). Twenty (12%) of the treated women had a medical infertility factor; for the rest, the reason to have assisted reproduction was social.

Sperm insemination in a natural cycle (without hormonal treatment) is less medically complicated, but has a poorer pregnancy outcome than regular IVF treatment. Ovulation stimulation takes place in order to induce physical ovulation in women with anovulation before intrauterine insemination (IUI), or as a step in regular IVF treatment. It is common to offer IVF treatment after, for example, two unsuccessful (natural or stimulated cycle) sperm inseminations.^26^ In the present study, 65.8% of the treated women underwent IVF treatment; however, the majority of these women had undergone IUI before proceeding to IVF treatment.

### Measurements

#### Demographic and medical data

The following demographic data were collected at T1: age, level of education, civil status, number of previous children, identity-release or known donation, pregnancy outcome at T2 and future reproductive plans at T3. In addition, the women could leave written comments about their future reproductive plans. Medical data, number of received treatments and length of relationship were collected from the medical record.

#### Analysis of dropout individuals between T1 and T2, and between T1 and T3

**Figure 1:**
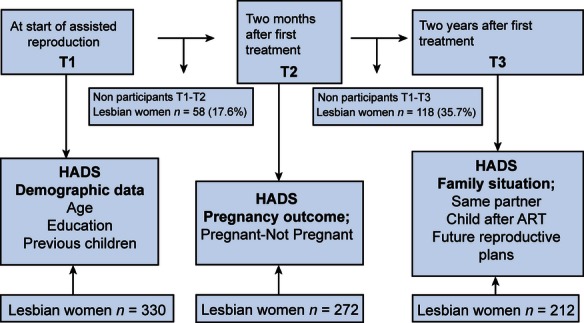
Flow chart of participants and nonparticipants during assisted reproductive treatment (ART). HADS, Hospital Anxiety and Depression Scale.

In a long-term prospective study such as this, over time participants drop out. Figure 1 presents an overview of participants and nonparticipants at each time point.

Furthermore, an analysis was performed in order to investigate the characteristics and possible reasons of those dropping out.

#### The Hospital Anxiety and Depression Scale (HADS)

To assess anxiety and symptoms of depression, HADS was used. HADS was developed by Zigmond and Snaith,^27^ in 1983, and is a self-assessment scale which has been found to be a reliable instrument for the detection of states of anxiety and depression in the setting of a hospital medical outpatient clinic. HADS comprises two subscales, one for anxiety symptoms and one for depression.^27^ For each subscale of seven items (each scored 0–3), a total score ranging from 0 to 21 can be obtained. A score of 0–7 for either subscale is regarded as being in the ‘normal’ range, a score of 8–10 is suggestive of the presence of mild levels of anxiety or depression, a score of 11–14 indicates moderate levels of anxiety or depression, and a score of 15–21 indicates severe levels of anxiety or depression; scores between 11 and 21 are regarded as clinically significant, i.e. the individual, when examined by an experienced mental health professional, would be highly likely to be diagnosed as suffering from an identifiable psychiatric disorder.^28^ In order to identify individuals with symptoms of anxiety or depression, the cut-off was set at eight or more, and cases and scores between 0 and 7 were defined as noncases.

#### Pregnancy outcome

At T2 and T3, the couples were asked to report pregnancy outcome, i.e. pregnant, not pregnant or if the pregnancy ended in a miscarriage.

#### Future reproductive plans

The third questionnaire contained questions about whether the participants were living with the same partner as at the commencement of treatment, as well as the couple's future reproductive plans: if they were planning to continue treatment, take a break from treatment, adopt a child or live without children. Six statements with four response alternatives each composed this questionnaire. The response alternatives were: ‘yes’, ‘maybe’, ‘no’ or ‘do not know’; in the present study, the response alternatives ‘maybe’ and ‘do not know’ have been merged. The couples were asked to respond to all of these six statements. Because of this, the response rate differed between the statements. In addition, open-ended comments were collected from 40 treated women and 36 partners.

### Data analysis

All statistical analysis was performed using IBM SPSS Statistics version 20. In all analyses, *P* < 0.05 was considered to be statistically significant. Chi squared test and Fisher's exact test were used to compare differences between the treated woman and her partner. Data collected in open-response format were categorized according to content. To illustrate and enrich the results, quotes from participants are presented.

## Results

### Demographic background data

Demographic and medical data are displayed in Table 1. The treated women were slightly younger than the partners, with a mean age of 32.12 years for the treated women and 33.46 years for the partners (*P* = 0.018). There were more treated women with a university degree than amongst the partners (*P* = 0.010). Both the treated women and the partners had previous children. The mean length of the relationship was 5.5 years for both treated women and the partners, ranging between 1 and 19 years, with a median of 5 years.

**Table 1:** Characteristics of lesbian women

	Lesbian couples (*n* = 330)		
	
	Treated (*n* = 165)	Partner (*n* = 165)	*P*
Age (years), mean (SD)	32.12 (±3.96)	33.46 (±5.96)	0.018
**Education**	***n* (%)**	***n* (%)**	
<12 years	54 (32.7)	81 (49.0)	0.010
University	111 (67.3)	84 (51.0)	
**Previous biological children**			
No	160 (97.0)	138 (83.6)	<0.001
Yes	5 (3.0)	27 (16.4)	
Same partner*			
No	11 (10.2)	10 (9.6)	
Yes	97 (89.8)	94 (90.4)	

* Living with the same partner at T3 (i.e. at follow-up at 2–5 years after treatment) as at inclusion in study (T1). T3, *n* = 108 treated women, *n* = 104 partners.

### Analysis of dropout individuals between T1 and T2 and between T1 and T3

Attrition analysis, comparing women who dropped out between T1 and T2 (*n* = 58, 17.6%) with women participating at T2 (*n* = 272), and women who dropped out between T1 and T3 (*n* = 118, 35.8%) with women participating at T3 (*n* = 212), showed no significant group differences with regard to sociodemographic data, pregnancy outcome or HADS scores.

### Anxiety and depressive symptoms

HADS scores are displayed in Table 2. Few women in the lesbian couples reported symptoms of anxiety and depression throughout the period of ART. A higher percentage of the treated women, compared with the partners, reported symptoms of anxiety at T2 (14% versus 5%, *P* = 0.011) and T3 (10% versus 4%, *P* = 0.018); as well as with symptoms of depression at T2 (4% versus 0%, *P* = 0.03) and T3 (3% versus 0%, *P* = 0.035).

**Table 2:** Comparison of Hospital Anxiety and Depression Scale (HADS) scores at the three time points and between treated women and the partners

	Anxiety T1	Anxiety T2	Anxiety T3	Depression T1	Depression T2	Depression T3

**Treated women**	***n* = 163**	***n* = 135**	***n* = 104**	***n* = 165**	***n* = 135**	***n* = 106**
**HADS score**						
0–7	150 (91.0)	116 (70.3)	87 (52.7)	165	130 (78.8)	101 (61.2)
8–10	7 (4.2)	10 (6.1)	11 (6.7)	0	3 (1.8)	3 (1.8)
11–14	2 (2.4)	7 (4.2)	5 (3.0)	0	2 (1.2)	2 (1.2)
15–21	2 (1.2)	2 (1.2)	1 (0.6)	0	0	0
Mean (SD)	4.09 (±2.86)	4.14 (±3.46)	4.31 (±3.55)	1.46 (±1.52)	2.22 (±2.41)	2.79 (±2.46)
Median	4	3	4	1	1	2
Range	0–15	0–19	0–17	0–7	0–13	0–12
Paired *t*-test between time points (A, anxiety; D, depression)		A1–A2, *P* = 0.150	A2–A3, *P* = 0.561 A1–A3, *P* = 0.044		D1–D2, *P* < 0.001	D2–D3, *P* = 0.900 D1–D3, *P* = 0.025

**Partners**	***n* = 162**	***n* = 135**	***n* = 101**	***n* = 162**	***n* = 135**	***n* = 99**
**HADS score**						
0–7	146 (88.5)	128 (77.6)	95 (57.6)	160 (97.0)	135 (81.8)	99 (60.0)
8–10	11 (6.7)	4 (2.4)	4 (2.4)	2 (1.2)	0	0
11–14	5 (3.0)	2 (2.1)	2 (1.2)	0	0	0
15–21	0	1 (0.6)	0	0	0	0
Mean (SD)	3.82 (±2.81)	3.41 (±2.79)	3.22 (±2.53)	1.66 (±1.59)	1.45 (±1.74)	2.37 (±1.85)
Median	3	3	3	1	1	2
Range	0–14	0–16	0–11	0–8	0–7	0–7
Paired *t*-test between time points (A, anxiety; D, depression)		A1–A2, *P* = 0.029	A2–A3, *P* = 0.818 A1–A3, *P* = 0.065		D1–D2, *P* = 0.014	D2–D3, *P* = 0.002 D1–D3, *P* = 0.370
Treated woman/partner*	A1, *P* = 0.342	A2, *P* = 0.011	A3, *P* = 0.015	D1, *P* = 0.248	D2, *P* = 0.030	D3, *P* = 0.035

* Because of the few cases, treated women and the partners were compared based on their HADS scores (0–7 versus 8–21) using *χ*^2^ test and Fisher's exact test.

An analysis comparing symptoms of anxiety and depression between the three time points was performed. For the treated women, an increase in anxiety scores was found between T1 and T3, and an increase in depression scores between T1 and T2/T3. Among the partners, anxiety scores decreased between T1 and T2, and depression scores increased between T1 and T2 and between T2 and T3. At T3, when the treatment was terminated, only five of the treated lesbian women reported symptoms of depression. Consequently, because of the few women with symptoms of depression, no relationships between demographic data, pregnancy outcome, future reproductive plans and symptoms of anxiety and depression were found.

### Pregnancy outcome

The second questionnaire included questions about the couple's current situation. Twenty three (13.9%) women reported a pregnancy after the first treatment and another 32 (30.9%) reported being pregnant at T2. Ten women had a miscarriage. Sixty couples were planning continuous treatment (see Table 3). The question about pregnancy outcome was repeated at T3 and, finally, 77 treated women (72.6%) had given birth to a child after ART (see Table 4).

**Table 3:** Pregnancy outcome in treated women after first and second treatment, time point 2 (T2)

	Couples at T2 (*n* = 136)	
	
	*n*	%
**Pregnancy outcome after first treatment**		
Pregnant	23	16.9
**Pregnancy outcome after second treatment**		
Pregnant	51	37.5
Plan new ART	60	44.1
Other	23	16.9

ART, assisted reproductive treatment.

**Table 4:** Future reproductive plans at time point 3 (T3)

	Couples (*n* = 106)	
	
	*n*	%
**Child after treatment, T3**		
Yes	77	72.6*
**Try new**		
Yes	35	40.7
Maybe/Do not know	28	32.5
No	23	26.7
**Try other medical treatment**		
Yes	4	6.9
Maybe/Do not know	12	20.6
No	42	72.4
**Take a break from treatment**		
Yes	7	14.6
Maybe/Do not know	15	31.2
No	26	54.2
**Discontinue treatment**		
Yes	3	6.5
Maybe/Do not know	9	19.6
No	34	73.9
**Adopt a child**		
Yes	2	3.8
Maybe/Do not know	11	21.2
No	39	75.0
**Live without children**		
Yes	1	3.4
Maybe/Do not know	6	20.6
No	22	75.9

* Counted from the 106 couples that responded to the third questionnaire.

### Future plans

The couple's future reproductive plans are displayed in Table 4. Forty per cent of the couples reported that they were planning continuous treatment and 54% reported that they were not planning to take a break from treatment. It was noteworthy that only three couples planned to discontinue treatment and only one couple stated that they planned to live without children. Two couples were considering adoption. Forty treated women and 36 partners wrote comments about their future plans. Of these, 21 treated women and 21 partners were identified as being from the same couple (55%). The open ended comments resulted in the identification of five main categories: (i) satisfied with the children we have got, six treated women/eight partners; (ii) ongoing treatment/pregnant/recently given birth, 12 treated women/12 partners; (iii) partner/co-mother treatment, nine treated women/five partners; (iv) continue treatment later, sibling treatment, frozen eggs at the clinic, 10 treated women/five partners; (v) no more treatment in Sweden – we are going to Denmark for continuous treatment, two treated women/two partners.

T3 also included questions about the couple's current cohabiting situation, and 11 couples (10.2%) reported that they were no longer cohabiting with the same partner as at T1.

## Discussion

### Main findings

In this study of Swedish lesbian women treated with sperm donation, the vast majority reported no symptoms of anxiety or depression.

### Strengths and weaknesses

This study has its limitations. Longitudinal studies tend to lose participants over time.^29^,^30^ This was also the case in this study, where the response rate dropped to 82.4% at T2 and to 64% at T3, 2–5 years after study inclusion. A response rate of 65% has been mentioned as acceptable for studies with self-completion postal questionnaires (which were used at T2 and T3).^29^,^30^ Although the sample size at T3 is somewhat low, these longitudinal data from a group of lesbian couples starting a family are unique. We believe that the findings in this study are valuable and add important knowledge about the psychological health in this growing group of patients in obstetric care.

Another weakness in this study is the limited knowledge about the individuals who dropped out. Our analysis of dropout individuals did not result in any information that would explain this, and it is difficult to speculate about the characteristics and circumstances of the women who dropped out.

HADS has been reported to demonstrate commendable psychometric validity and reliability, Cronbach's alpha (anxiety, 0.80–0.93; depression, 0.81–0.90),^31^,^32^ and has been used previously in a prospective longitudinal study^28^; this provides strength to the results of the present study.

### Interpretation

Psychological health in couples undergoing IVF treatment has been studied frequently,^24^,^33^ as have psychological health issues in lesbian women^15^,^16^,^34^ and lesbian women trying to conceive.^10^,^18^,^20^,^35^ However, longitudinal psychological health in lesbian women undergoing assisted reproduction with sperm donation treatment has not been studied in detail. The present results of an increase in anxiety among treated women at T2 are in line with the fact that undergoing IUI with donated sperm, as well as IVF treatment *per se*, is associated with increased anxiety.^36^ Although antenatal anxiety has been associated with the development of depression,^14^,^37^ in our study only five treated women had symptoms of depression on follow-up at T3.

The legal and social recognition of homosexuals has been suggested to offer a positive, protective and moderating effect to minority stress^38^,^39^ and to improve psychological health in lesbian women.^10^,^11^,^20^ In Sweden, equality in federal, legal and social contexts exists between homosexuals and heterosexuals. Marriage, access to free assisted reproduction within the national healthcare system and the co-mothers equal parental status in law are domestic protections that benefit homosexual couples. The small number of women with symptoms of anxiety and depression in this study may be a result of the legal and social acceptance of homosexuality in Sweden. By virtue of the fact that the lesbian couples decided to start a family with children suggests that they are probably psychologically healthy and satisfied with their relationships/marriages.^1^ Relationship and marital satisfaction has been found to be important to psychological wellbeing in many studies.^40^,^41^ Building a joint family and going through sperm donation treatment are deep and life-long commitments to lesbian couples and may instil a sense of commitment to the couples.

One can assume that, as the lesbian women in our study were cohabiting in committed relationships, they were disclosed, and this may have contributed to the psychological wellbeing of the women in this study. ‘To be out’ has been described as being associated with psychological health in lesbian women^15^ and, moreover, lesbians who are out are more likely to align with friends and to receive social support.^16^,^42^ The desires to have children were positively illustrated in the lesbian women's open-ended comments. Spirits of joy and satisfaction about their ongoing family forming characterised the lesbian women's comments.

In this study, 11 couples reported that they had divorced or separated since they had commenced treatment. We are unaware of whether or not the number of couples that divorced during this time is an expression of poor relationship quality and satisfaction; unfortunately, we did not ask for the reasons for divorce. In a study of the demographics of same-sex marriage in Norway and Sweden, it was stated that patterns in divorce risks are rather similar in same-sex and opposite-sex marriages, but divorce risk levels are considerably higher in same-sex marriages.^43^ Further studies are essential to understand relationship breakup in lesbian couples.

## Conclusion

This study reports good psychological health in lesbian couples undergoing assisted reproduction with donated sperm to start a family. The small number of participants presenting with symptoms of anxiety and depression suggests that the medical and psychosocial investigation accomplished by infertility clinics is solid and careful. Future long-term studies should address psychological aspects in lesbian families with children, as well as psychological health in lesbian couples with unsuccessful treatment.
